# Interfacial undercooling in solidification of colloidal suspensions: analyses with quantitative measurements

**DOI:** 10.1038/srep28434

**Published:** 2016-06-22

**Authors:** Jiaxue You, Lilin Wang, Zhijun Wang, Junjie Li, Jincheng Wang, Xin Lin, Weidong Huang

**Affiliations:** 1 State Key Laboratory of Solidification Processing, Northwestern Polytechnical University, Xi’an 710072, P.R. China; 2 School of Materials Science and Engineering, Xi’an University of Technology, Xi’an 710048, P.R. China

## Abstract

Interfacial undercooling in the complex solidification of colloidal suspensions is of significance and remains a puzzling problem. Two types of interfacial undercooling are supposed to be involved in the freezing of colloidal suspensions, i.e., solute constitutional supercooling (SCS) caused by additives in the solvent and particulate constitutional supercooling (PCS) caused by particles. However, quantitative identification of the interfacial undercooling in the solidification of colloidal suspensions, is still absent; thus, the question of which type of undercooling is dominant in this complex system remains unanswered. Here, we quantitatively measured the static and dynamic interface undercoolings of SCS and PCS in ideal and practical colloidal systems. We show that the interfacial undercooling primarily comes from SCS caused by the additives in the solvent, while PCS is minor. This finding implies that the thermodynamic effect of particles from the PCS is not the fundamental physical mechanism for pattern formation of cellular growth and lamellar structure in the solidification of colloidal suspensions, a general case of ice-templating method. Instead, the patterns in the ice-templating method can be controlled effectively by adjusting the additives.

The solidification of colloidal suspensions is commonly encountered in a variety of natural processes such as the growth of sea ice^1^ and frost heave^2^, and engineering situations such as cryobiology^3^, tissue engineering^4^, ice-templating bio-inspired porous materials and composites^5^,^6^,^7^,^8^,^9^,^10^,^11^,^12^,^13^,^14^,^15^,^16^,^17^,^18^,^19^, thermal energy storage^20^ and soil remediation^21^. In particular, ice-templating porous materials have attracted increasing attention due to the novel micro-aligned structures that can be easily produced for a wide range of applications^5^,^6^,^7^,^8^,^9^,^10^,^11^,^12^,^13^,^14^,^15^,^16^,^18^.

One of the key issues therein is the pattern formation. The formation of microstructures is closely related to the interfacial instability during the freezing of colloidal suspensions and the subsequent development of interfacial morphologies^22^,^23^. The interfacial instability during freezing strongly depends on the interfacial undercooling, which has been extensively revealed by the research community in studies of solidification^24^,^25^. Accordingly, it is believed that the interfacial undercooling is also of significance in microscopic pattern formation of freezing colloidal suspensions.

Two types of interfacial undercooling have been proposed to occur in the solidification of colloidal suspensions, i.e., solute constitutional supercooling (SCS) caused by additives in the solvent^12^,^13^,^17^, and particulate constitutional supercooling (PCS) caused by particles^22^,^23^. The theory of SCS is based on the classical alloy solidification principle^12^,^24^,^25^, while the theory of PCS is derived from multi-particle thermodynamics^22^, a typical characteristic of the colloidal suspensions system. In the past decade, since its initial proposal, the PCS theory has attracted the extensive attention of researchers in many fields, such as porous ceramics^26^, polymers and composites^27^,^28^, bone tissue engineering^29^, metal–ceramic composites^30^, the science of soft matter^31^,^32^, geophysical science^33^, thermal energy storage^34^, crystal growth^35^, cryobiology^36^, etc. To date, there has been no report on the quantitative measurements of interfacial undercooling during the solidification of colloidal suspensions, much less on the distinctions between these two types of interfacial undercooling.

The question of which type of undercooling is dominant in the solidification of colloidal suspensions remains an unsolved but important issue. First, determining the individual effects of SCS and PCS can improve the fundamental understanding of the physical mechanism of pattern formation during the freezing of colloidal suspensions. Moreover, this determining the individual effects will further pave the way toward controlling the microscopic pattern formation in this complex system. For example, if the PCS is dominant, then the freezing pattern can be modulated *via* the particle size or particle shape; otherwise, it can be adjusted by changing the additives. Here, we quantitatively measured the static and dynamic interfacial undercoolings of SCS and PCS in ideal and practical colloidal systems.

In this letter, interfacial undercoolings were quantitatively measured based on a novel experimental method. The contributions of SCS and PCS were confirmed for the first time. A detailed description of the experimental apparatus and gauging method is given in ref. 37. A sketch of this method is shown in Fig. S1 (Supplementary Information). In the method, the interfacial undercooling is visualized through the discrepancy of solid/liquid interfacial positions in two adjacent Hele-Shaw cells of the colloidal suspension and its compared counterpart in a uniform thermal gradient apparatus. The thermal gradient plays a key role on measuring interfacial undercoolings and positions. In order to reduce radiative and convective perturbations, the thermal blocks were covered with heat-insulating shield and the gap between hot and cold copper blocks was covered with double-glazed windows, which had both excellent thermal insulation and optical microscopic observation. More importantly, these two Hele-Shaw cells (the composition of their walls is glass, with a cross-section of 2 mm × 0.1 mm) were placed tightly on a sufficiently large plane glass plate (with a cross-section of 30 mm × 0.2 mm) in order to obtain a fixed linear thermal gradient. The thermal gradient in Hele-Shaw cells is determined by heat conduction of the plane glass plate so as to minimize the effect of the difference in the thermal conductivities of particles, liquid water and ice on the measurements of interfacial undercoolings. The validity of the measurements is verified based on tests with different thermal gradients. The SCS and PCS can be well distinguished by designing different compared counterparts. After quantitatively measuring the SCS and PCS in different systems of colloidal suspensions, we analyzed the results based on theoretical predictions. In the quantitative measurements, ideal systems of polystyrene microsphere (PS) suspensions were first considered. After discovering the minor contribution of PCS, we also measured the interfacial undercooling in practical systems of α-alumina suspensions with both static and dynamic interface to further confirm the contributions of SCS and PCS.

The first system we chose is PS suspensions (Bangs Lab, USA). The nominal solvent of PS suspensions is deionized water. The density of PS particles is almost the same as that of water. The mean diameter of the particles is d = 1.73 μm with a poly-dispersity smaller than 5%, and the initial volume fraction of particles is ϕ_0_ = 33%. The PS suspensions system is stable only with weak sedimentation, i.e., it is an ideal system to investigate the freezing of colloidal suspensions. Although the solvent of deionized water is marked on the nominal label of PS suspensions, we believe that there are still very small quantities of residual solutes from the synthesis of PS particles, even after great efforts of purification in these commercial PS suspensions. The residual solutes will also cause SCS during the freezing of PS suspensions. Therefore, in the measurement, first, we verified this type of SCS by comparing deionized water with the supernatant from the PS suspensions by centrifugation. Furthermore, we compared each PS suspension with its supernatant to confirm the individual contribution of PCS. The combination of SCS and PCS accounts for the whole interfacial undercooling during the solidification of colloidal suspensions.

Figure 1(a) shows the measurement of SCS through the interface position comparison between the deionized water (left cell of Fig. 1(a)) and the supernatant (right cell of Fig. 1(a)) within a microscopic image. The upper end of the cell is the heating zone, while the lower end of the cell is the cooling zone, which builds a linear thermal gradient G = 7.23 K/cm. The pulling speed V is 0. In Fig. 1(a), the position of solid/liquid interface in the cell of deionized water is much higher than that of the supernatant, which indicates that the freezing point of the deionized water is much higher than that of the supernatant. The discrepancy of the solid/liquid interface positions between the deionized water and the supernatant is 171 μm, corresponding a SCS of 0.123 K with G = 7.23 K/cm.

Comparison of the interfacial position between the colloidal suspension and its supernatant is shown in Fig. 1(b), which exhibits the measurement of PCS. The interfacial position of the supernatant is almost identical to that of its suspension, which means that the freezing point of the supernatant is almost the same as that of its suspensions. Therefore, the PCS is almost undetectable and should be smaller than 0.01 K if it exists in this PS colloidal suspensions system (the precision of the experimental method has been demonstrated to be 0.01 K^37^).

Consequently, in Fig. 1, the interfacial undercooling of colloidal suspensions mainly comes from SCS. To further confirm this conclusion, the interfacial undercoolings of PS suspensions systems with particles of different diameters and different volume fractions were measured. All the results are similar to that in Fig. 1. The comparisons of interface positions are shown in Fig. S2 (Supplementary Information), and the interfacial undercoolings of SCS and PCS are shown in Table 1. Surprisingly, all the results indicate that the PCS makes minor contribution to the interfacial undercooling of PS colloidal suspensions. However, a 5K PCS was reported in ref. 23 under d = 1 μm and ϕ_0_ = 50%, which were similar to our test conditions, d = 1.73 μm and ϕ_0_ = 33%. These two testing results (5 K and less than 0.01 K) are obviously divergent.

These unexpected results deserve further analysis in considering the PCS theory^22^,^23^,^38^,^39^. Recently, PCS theory was first proposed and applied to address some puzzling phenomena with numerous unexplained features in freezing colloidal suspensions^40^,^41^. In PCS theory, the particle-controlled interfacial undercooling is described as


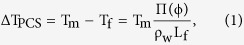


where 
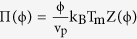
 is the osmotic pressure caused by the concentrated layer of particles, and 
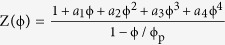
 is the dimensionless compressibility factor. T_m_ is the melting point of ice. T_f_ is the depressed melting point. ρ_w_ is the density of water. L_f_ is the latent heat, and ϕ is the volume fraction of particles. k_B_ is the Boltzmann constant and 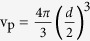 is the volume of a particle. The maximum volume fraction of particles is ϕ_p_ = 0.64, 0 ≤ ϕ ≤ 0.64. *a*_1_, *a*_2_, *a*_3_ and *a*_4_ are fitting parameters of П. In this theory, the PCS comes from the depressed equilibrium melting point caused by the osmotic pressure of concentrated particles ahead of the freezing interface, which includes consolidated physical foundations^38^. However, the determination of the dimensionless compressibility factor in osmotic pressure is casual in refs 23 and 39. The variation of *Z*(ϕ) with different ϕ has been well investigated^42^,^43^. Originally, the fitting parameters from refs 22,38,43 and 44 are


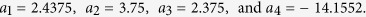


However, the fitting parameters used in refs 23 and 39 were


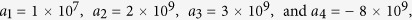


in order to give way to the experimental results of filtration pressure of drilling fluid filtercakes^23^. Subsequently, experimental depressions of the freezing point were used to confirm these huge fitting parameters. However, the experimental data of depressed freezing points cannot be used to confirm the predicted PCS. First, the colloidal suspensions contained a large number of ions which can dramatically depress the freezing point of water^45^. Second, the bentonite used was a mixture of a variety of particles with different sizes, while П greatly depends on the particle radius, with an inverse proportion to the third power of the particle radius and thus PCS is inversely proportional to the third power of the particle radius.

By using the original fitting parameter for Z(ϕ), the theoretical PCS in the PS systems investigated here is approximately 10^−9^ K, shown as prediction A in Table 1, which is too small to be detected. Both these theoretical results and the present measurements demonstrated that the PCS is minor compared with the SCS.

In PCS theory, the PCS is inversely proportional to the third power of the particle radius. Moreover, because the α−alumina suspensions are quite commonly used in the preparing of ice-templating porous ceramics, the interfacial undercoolings in the freezing of α−alumina colloidal suspensions were further measured. The α−alumina powder with a mean diameter d = 50 nm and a density of 3.97 g cm^−3^ are used (Wanjing New Material, Hangzhou, China, ≥99.95% purity, monodispersity). The alumina suspensions were prepared by using HCl (hydrogen chloride) and deionized water as the solvent following ref. 46. Initial volume fractions were ϕ_0_ = 2.72%, 3.63%, 9.74% and 20.12% (wt% = 10, 13, 30 and 50) in four different systems, respectively. These measurements were also made under the conditions of thermal gradient G = 7.23 K/cm and pulling speed V = 0. The SCS and PCS from the measurements are shown in Fig. 2. The interface position comparisons of the static SCS and PCS for alumina suspensions are shown in Fig. S3 (Supplementary Information). The measured PCS is still extremely small (blue triangular points in Fig. 2), i.e., it makes minor contribution to the total interfacial undercoolings compared with the SCS, as shown in the inset of Fig. 2. We further designed different thermal gradients to test the PCS in an identical system so as to confirm that the coincidence of interface positions indicates the coincidence of interface temperatures, although the difference in thermal conductivities of alumina suspension and its supernatant may affect the local thermal gradient and the interfacial position. The measured PCS under different thermal gradients keeps the same, as shown in Fig. S4 (Supplementary Information). The theoretical prediction of PCS is approximately 10^−6^ K (prediction A in Fig. 2 and Table 2), which is still undetectable in the present setup, but, consistent with our experimental data. Only in the extreme case, e.g., the particles of d = 1 nm and ϕ ≈ ϕ_p_ were used, the PCS could be comparable to SCS. However, in most cases, the PCS’s contribution to the interfacial undercooling is minor compared with the SCS from the solvent in the solidification of colloidal suspensions.

In the above measurements, the static interfacial undercoolings were clarified. The dynamic interfacial undercooling during the freezing of colloidal suspensions, another important aspect related to the pattern formation, has never been reported before. Our experimental apparatus can also be used to quantitatively identify the dynamic interfacial undercooling^37^. Here, we measured the dynamic interfacial undercooling in the alumina suspensions of d = 50 nm, ϕ_0_ = 3.63% to further reveal the contribution of SCS and PCS. The comparison of the colloidal suspension to its supernatant can reveal the dynamic PCS, and the comparison of deionized water to the supernatant can reveal the dynamic SCS.

Figure 3 shows the steady dynamic interface positions of the supernatant, deionized water and colloidal suspension under V = 8.217 μm/s and G = 7.23 K/cm. The steady state of dynamic PCS was verified as shown in Movie S1 (Supplementary Information). The comparison between the interfacial positions of the supernatant and deionized water indicates a dynamic SCS of 0.09K, as shown in Fig. 3(a). However, Fig. 3(b) shows that the dynamic PCS is undetectable, although the particles have accumulated in front of the advancing freezing interface and formed an obvious concentrated layer. Similar to the static case, the dynamic PCS is also minor compared with the obvious dynamic SCS. Therefore, the concentrated particle layer seems unable to cause an obvious PCS.

Even with different pulling speeds, the results regarding dynamic PCS are similar to that shown in Fig. 3 (shown in Supplementary Information, Fig. S5). The concentrated layer of particles in front of the freezing interface scarcely causes dynamic PCS under different pulling speeds. In contrast, the dynamic SCS varies with the pulling speed. To reveal the dynamic SCS, the interfacial position comparisons are shown in Fig. S6 (Supplementary Information). Fig. 4 shows the variation in the dynamic SCS with different pulling speeds. The increase in the dynamic SCS with decreased pulling speed is consistent with the classical alloy solidification principle^47^. This result indicates that, in the dynamic case, the SCS still plays a dominant role compared with the minor dynamic PCS for cases of different pulling speeds.

Based on the above systematic measurements, the PCS is minor in both static and dynamic cases; in contrast, the effect of SCS caused by additives in the solvent is dominant. Accordingly, the thermodynamic effect of particles from the PCS is not the fundamental physical mechanism for cellular growth and lamellar structure that are associated with the solidification of colloidal suspensions^48^ which inevitably contains a large number of solutes, especially in the ice-templating method. Nevertheless, when the effect of solutes is absent, some other effects such as the force interactions between particles and freezing interface perhaps should be considered to reveal the pattern of intermittent lenses^48^. The present experimental results clearly demonstrate that the effects of additives are dominant in the ice-templating process^12^,^13^,^17^,^48^.

## Conclusions

We considered the puzzling phenomenon of interfacial undercoolings in the solidification of colloidal suspensions *via* quantitative measurements of solute constitutional supercooling (SCS) and particulate constitutional supercooling (PCS). Based on systematic quantitative experimental measurements of both static and dynamic cases within different systems of colloidal suspensions, we found that the interfacial undercooling mainly comes from SCS caused by the additives in the solvent, while the PCS is minor. The results imply that the thermodynamic effect of particles from the PCS is not the fundamental physical mechanism for pattern formation of cellular growth and lamellar structure that are associated with the solidification of colloidal suspensions. These fundamental findings can greatly enhance our understanding of the physics of freezing colloidal suspensions in the ice-templating method, a general method to produce novel and advanced biomaterials as well as multifunctional-materials, and pave the way toward controlling pattern formation in freezing colloidal suspensions^48^.

## Additional Information

**How to cite this article**: You, J. *et al.* Interfacial undercooling in solidification of colloidal suspensions: analyses with quantitative measurements. *Sci. Rep.* **6**, 28434; doi: 10.1038/srep28434 (2016).

## Supplementary Material

Supplementary Information

Supplementary Movie S1

## Figures and Tables

**Figure 1:**
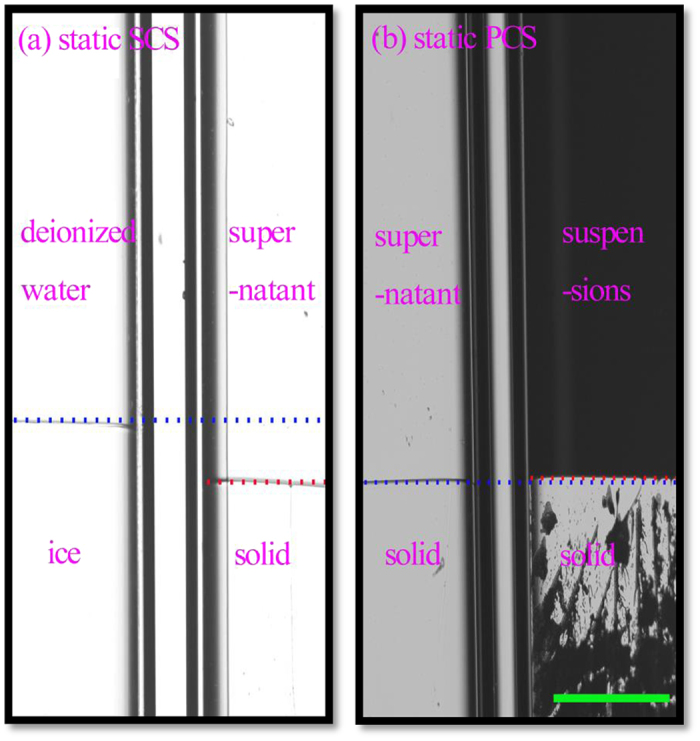
The static interfacial positions in two side-by-side Hele-Shaw cells of the deionized water and the supernatant from PS colloidal suspensions of d = 1.73 μm, ϕ_0_ = 33% (**a**); and two side-by-side Hele-Shaw cells of the colloidal suspensions and its supernatant (**b**) in a uniform thermal gradient of G = 7.23 K/cm. The distances between the static interfacial positions reveal the static interfacial undercoolings. The pulling speed is V = 0. The scale bar is 200 μm.

**Figure 2:**
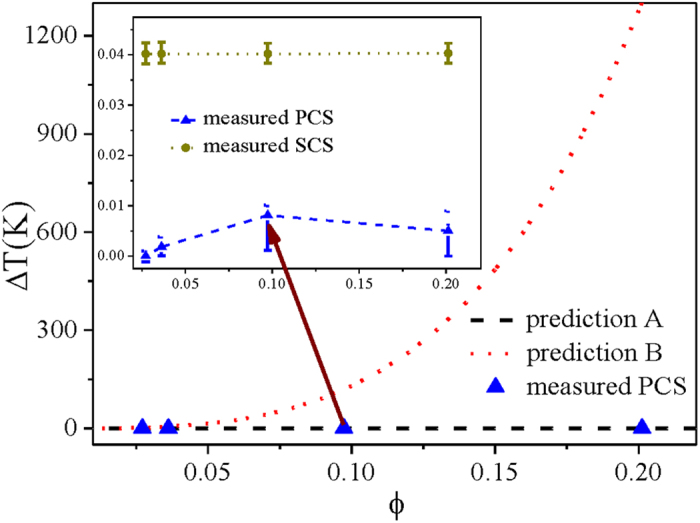
Measured PCS compared with the theoretical PCS. Prediction A of PCS is from refs 22 and 38, while prediction B of PCS comes from refs 23 and 39. The inset is the measured value of SCS and PCS for alumina suspensions with d = 50 nm under G = 7.23 K/cm and V = 0.

**Figure 3:**
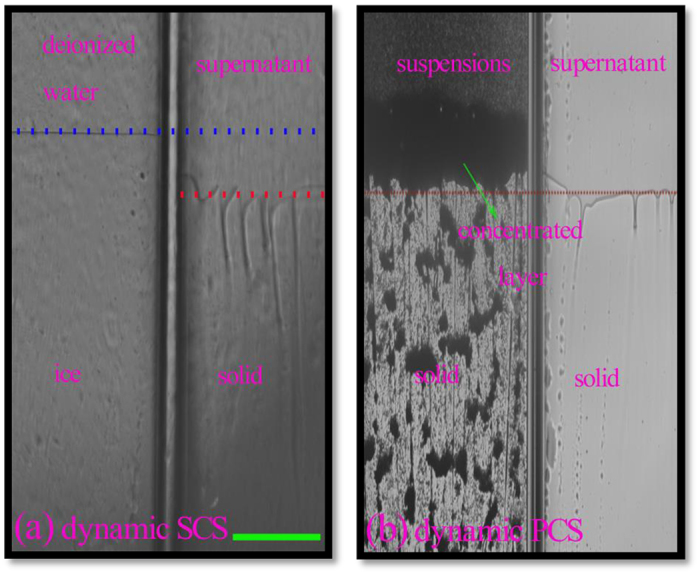
The steady-state interfacial positions in two side-by-side Hele-Shaw cells of the deionized water and the supernatant from alumina suspensions of d = 50nm, ϕ_0_ = 3.63% (**a**); and two side-by-side Hele-Shaw cells of the alumina suspensions and its supernatant (**b**) in a uniform thermal gradient of G = 7.23 K/cm. The distances between the steady-state interfacial positions reveal the dynamic interfacial undercoolings. The pulling speed is V = 8.217 μm/s. The scale bar is 200 μm.

**Figure 4:**
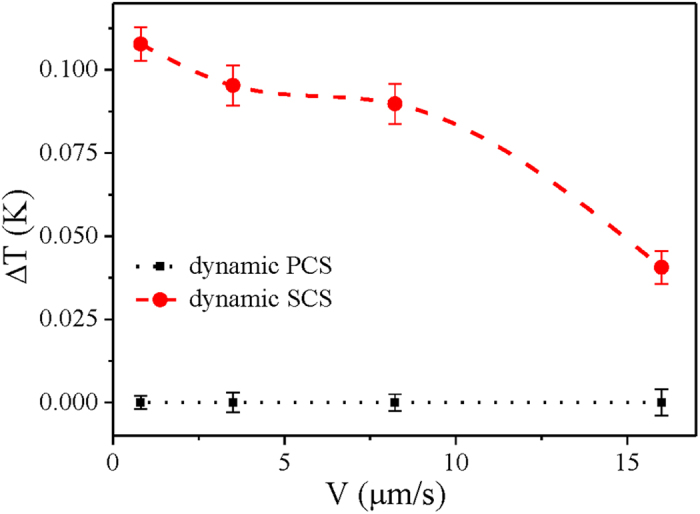
Measured dynamic SCS and PCS for alumina suspensions with d = 50 nm, ϕ_0_ = 3.63% under different pulling speeds; G = 7.23 K/cm.

**Table 1:** Static undercoolings from measurements and predictions.

	PS colloidal suspensions			
d (μm)	1		1.73	
ϕ_0_	20%		20%	33%
Measured SCS (10^−2^ K)	12.1 ± 0.5		12.3 ± 0.4	12.3 ± 0.4
Measured PCS (10^−2^ K)	0 ± 0.18		0 ± 1.87	0 ± 0.16
PCS theoretical predictions (10^−2^ K)	A	3.06 × 10^−7^	5.91 × 10^−8^	1.69 × 10^−8^
	B	17.2	3.34	19.5

Prediction A is from refs 22 and 38, while prediction B comes from refs 23 and 39. For PS colloidal suspensions of different d and ϕ_0_.

**Table 2:** Static undercoolings from measurements and predictions.

	alumina suspensions				
d (μm)	0.05				
ϕ_0_	2.72%		3.63%	9.74%	20.12%
Measured SCS (10^−2^ K)	4.01 ± 0.42		4.02 ± 0.42	4.03 ± 0.42	4.02 ± 0.42
Measured PCS (10^−2^ K)	0 ± 0.2		0.18 ± 0.22	0.91 ± 0.85	0 ± 0.90
PCS theoretical predictions (10^−2^ K)	A	1.93 × 10^−4^	6.98 × 10^−4^	7.45 × 10^−4^	2.24 × 10^−3^
	B	260	1640	12830	127701

Prediction A is from refs 22 and 38, while prediction B comes from refs 23 and 39. For alumina suspensions of different ϕ_0_.

## References

[b1] Vancoppenolle M. *et al.* Role of sea ice in global biogeochemical cycles: emerging views and challenges. Quaternary Science Reviews 79, 207–230 (2013).

[b2] Peppin S. S. L. & Style R. W. The Physics of Frost Heave and Ice-Lens Growth. Vadose Zone Journal 12 (2013).

[b3] Mazur P. Freezing of living cells: mechanisms and implications. Vol. 247 (1984). 10.1152/ajpcell.1984.247.3.C125 6383068

[b4] Wegst U. G. K., Schecter M., Donius A. E. & Hunger P. M. Biomaterials by freeze casting. Vol. 368 (2010). 10.1098/rsta.2010.0014 20308117

[b5] Deville S., Saiz E., Nalla R. K. & Tomsia A. P. Freezing as a Path to Build Complex Composites. Science 311, 515–518 (2006). 16439659 10.1126/science.1120937

[b6] Deville S., Saiz E. & Tomsia A. P. Ice-templated porous alumina structures. Acta Materialia 55, 1965–1974 (2007).

[b7] Deville S. Freeze-Casting of Porous Ceramics: A Review of Current Achievements and Issues. Advanced Engineering Materials 10, 155–169 (2008).

[b8] Deville S. *et al.* *In Situ* X-Ray Radiography and Tomography Observations of the Solidification of Aqueous Alumina Particles Suspensions. Part II: Steady State. Journal of the American Ceramic Society 92, 2497–2503 (2009).

[b9] Lasalle A., Guizard C., Maire E., Adrien J. & Deville S. Particle redistribution and structural defect development during ice templating. Acta Materialia 60, 4594–4603 (2012).

[b10] Deville S., Adrien J., Maire E., Scheel M. & Di Michiel M. Time-lapse, three-dimensional *in situ* imaging of ice crystal growth in a colloidal silica suspension. Acta Materialia 61, 2077–2086 (2013).

[b11] Deville S. Ice-templating, freeze casting: Beyond materials processing. Journal of Materials Research 28, 2202–2219 (2013).

[b12] Zhang H. *et al.* Aligned two- and three-dimensional structures by directional freezing of polymers and nanoparticles. Nat Mater 4, 787–793 (2005). 16184171 10.1038/nmat1487

[b13] Qian L. & Zhang H. Controlled freezing and freeze drying: a versatile route for porous and micro-/nano-structured materials. Journal of Chemical Technology & Biotechnology 86, 172–184 (2011).

[b14] Wegst U. G. K., Bai H., Saiz E., Tomsia A. P. & Ritchie R. O. Bioinspired structural materials. Nat Mater 14, 23–36 (2015). 25344782 10.1038/nmat4089

[b15] Bouville F., Maire E. & Deville S. Self-Assembly of Faceted Particles Triggered by a Moving Ice Front. Langmuir 30, 8656–8663 (2014). 24432973 10.1021/la404426d

[b16] Bouville F. *et al.* Strong, tough and stiff bioinspired ceramics from brittle constituents. Nat Mater 13, 508–514 (2014). 24658117 10.1038/nmat3915

[b17] Delattre B., Bai H., Ritchie R. O., De Coninck J. & Tomsia A. P. Unidirectional Freezing of Ceramic Suspensions: *In Situ* X-ray Investigation of the Effects of Additives. ACS Applied Materials & Interfaces 6, 159–166 (2014). 24341868 10.1021/am403793x

[b18] Bai H., Polini A., Delattre B. & Tomsia A. P. Thermoresponsive Composite Hydrogels with Aligned Macroporous Structure by Ice-Templated Assembly. Chemistry of Materials 25, 4551–4556 (2013). 24489436 10.1021/cm4025827 PMC3904501

[b19] Lasalle A. *et al.* Ice-Templating of Alumina Suspensions: Effect of Supercooling and Crystal Growth During the Initial Freezing Regime. Journal of the American Ceramic Society 95, 799–804 (2012).

[b20] Khodadadi J. M. & Hosseinizadeh S. F. Nanoparticle-enhanced phase change materials (NEPCM) with great potential for improved thermal energy storage. International Communications in Heat and Mass Transfer 34, 534–543 (2007).

[b21] Gay G. & Azouni M. A. Forced Migration of Nonsoluble and Soluble Metallic Pollutants ahead of a Liquid–Solid Interface during Unidirectional Freezing of Dilute Clayey Suspensions. Crystal Growth & Design 2, 135–140 (2002).

[b22] Peppin S. S., Worster M. G. & Wettlaufer J. Morphological instability in freezing colloidal suspensions. Proceedings of the Royal Society A: Mathematical, Physical and Engineering Science 463, 723–733 (2007).

[b23] Peppin S., Wettlaufer J. & Worster M. Experimental verification of morphological instability in freezing aqueous colloidal suspensions. Physical Review Letters 100, 238301 (2008). 18643549 10.1103/PhysRevLett.100.238301

[b24] Tiller W. A., Jackson K. A., Rutter J. W. & Chalmers B. The redistribution of solute atoms during the solidification of metals. Acta Metallurgica 1, 428–437 (1953).

[b25] Mullins W. W. & Sekerka R. F. Stability of a Planar Interface During Solidification of a Dilute Binary Alloy. Journal of Applied Physics 35, 444–451 (1964).

[b26] Waschkies T., Oberacker R. & Hoffmann M. J. Investigation of structure formation during freeze-casting from very slow to very fast solidification velocities. Acta Materialia 59, 5135–5145 (2011).

[b27] Zhang H. & Cooper A. I. Aligned porous structures by directional freezing. Advanced materials 19, 1529–1533 (2007).

[b28] Gun’ko V. M., Savina I. N. & Mikhalovsky S. V. Cryogels: Morphological, structural and adsorption characterisation. Advances in Colloid and Interface Science 187–188, 1–46 (2013). 10.1016/j.cis.2012.11.001 23218507

[b29] Xia Z. *et al.* Fabrication and characterization of biomimetic collagen–apatite scaffolds with tunable structures for bone tissue engineering. Acta Biomaterialia 9, 7308–7319 (2013). 23567944 10.1016/j.actbio.2013.03.038 PMC3738228

[b30] Piat R., Sinchuk Y., Vasoya M. & Sigmund O. Minimal compliance design for metal–ceramic composites with lamellar microstructures. Acta Materialia 59, 4835–4846 (2011).

[b31] Lowen H. Particle-resolved instabilities in colloidal dispersions. Soft Matter 6, 3133–3142 (2010).

[b32] French R. H. *et al.* Long range interactions in nanoscale science. Reviews of Modern Physics 82, 1887–1944 (2010).

[b33] Dash J. G., Rempel A. W. & Wettlaufer J. S. The physics of premelted ice and its geophysical consequences. Reviews of Modern Physics 78, 695–741 (2006).

[b34] El Hasadi Y. M. F. & Khodadadi J. M. One-dimensional Stefan problem formulation for solidification of nanostructure-enhanced phase change materials (NePCM). International Journal of Heat and Mass Transfer 67, 202–213 (2013).

[b35] Ivall J., Hachem M., Coulombe S. & Servio P. Behavior of Surface-Functionalized Multiwall Carbon Nanotube Nanofluids during Phase Change from Liquid Water to Solid Ice. Crystal Growth & Design 15, 3969–3982 (2015).

[b36] John Morris G. & Acton E. Controlled ice nucleation in cryopreservation‒A review. Cryobiology 66, 85–92 (2013). 23246475 10.1016/j.cryobiol.2012.11.007

[b37] You J. *et al.* *In situ* observation the interface undercooling of freezing colloidal suspensions with differential visualization method. Review of Scientific Instruments 86, 084901 (2015). 26329221 10.1063/1.4928108

[b38] Peppin S., Elliott J. & Worster M. Solidification of colloidal suspensions. Journal of Fluid Mechanics 554, 147–166 (2006).

[b39] Peppin S., Majumdar A. & Wettlaufer J. In Proceedings of the Royal Society of London A: Mathematical. Physical and Engineering Sciences. 466, 177–194 (2010).

[b40] Deville S. *et al.* Metastable and unstable cellular solidification of colloidal suspensions. Nature Materials 8, 966–972 (2009). 19898459 10.1038/nmat2571

[b41] Style R. W., Peppin S. S. L., Cocks A. C. F. & Wettlaufer J. S. Ice-lens formation and geometrical supercooling in soils and other colloidal materials. Physical Review E 84, 041402 (2011). 10.1103/PhysRevE.84.041402 22181141

[b42] Carnahan N. F. & Starling K. E. Equation of State for Nonattracting Rigid Spheres. The Journal of Chemical Physics 51, 635–636 (1969).

[b43] Shanti N. O., Araki K. & Halloran J. W. Particle redistribution during dendritic solidification of particle suspensions. Journal of the American Ceramic Society 89, 2444–2447 (2006).

[b44] Ferrar J. A. & Solomon M. J. Kinetics of colloidal deposition, assembly, and crystallization in steady electric fields. Soft matter 11, 3599–3611 (2015). 25797453 10.1039/c4sm02893g

[b45] Kozlowski T. Soil freezing point as obtained on melting. Cold regions science and technology 38, 93–101 (2004).

[b46] Anderson A. M. & Worster M. G. Periodic ice banding in freezing colloidal dispersions. Langmuir 28, 16512–16523 (2012). 23110707 10.1021/la303458m

[b47] Burden M. H. & Hunt J. D. Cellular and dendritic growth. II. Journal of Crystal Growth 22, 109–116 (1974).

[b48] Wang L., You J., Wang Z., Wang J. & Lin X. Interface instability modes in freezing colloidal suspensions: revealed from onset of planar instability. Scientific Reports 6, 23358 (2016). 26996630 10.1038/srep23358 PMC4800406

