# Supplementary material for: Interfacial undercooling in solidification of colloidal suspensions: analyses with quantitative measurements
Source: Sci Rep. 2016 Jun 22;6:28434. doi: 10.1038/srep28434 (PMC4916454; doi:10.1038/srep28434)
Supplement: Supplementary Information [file srep28434-s1.pdf]

Interfacial undercooling in solidification of colloidal suspensions: analyses with quantitative measurements

Jiaxue You[1], Lilin Wang[2], Zhijun Wang[1*], Junjie Li[1], Jincheng Wang[1*], Xin Lin[1] and Weidong Huang[1]

1-State Key Laboratory of Solidification Processing, Northwestern Polytechnical University, Xi'an 710072, P. R. China

2-School of Materials Science and Engineering, Xi'an University of Technology, Xi'an 710048, P. R. China

Supplementary Information

Figures S1-S6 and Movie S1

*Corresponding author. Tel.:86-29-88460650; fax: 86-29-88491484
E-mail address: zhjwang@nwpu.edu.cn (Zhijun Wang), jchwang@nwpu.edu.cn (Jincheng Wang)

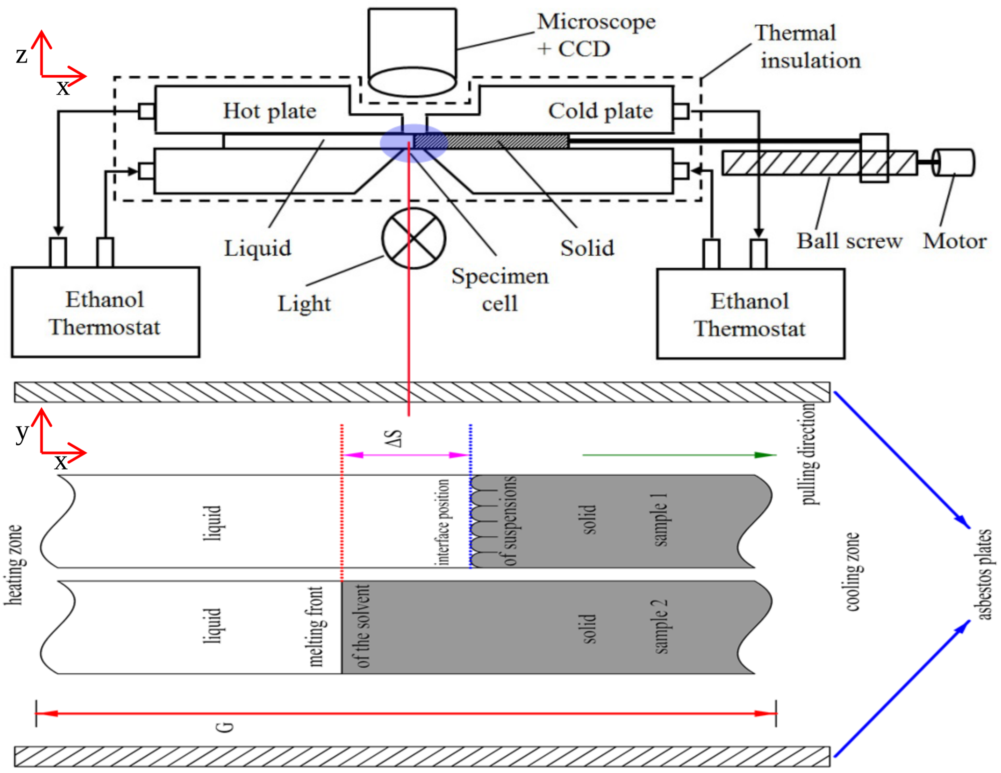

Fig.S1 Schematic of horizontal directional freezing stage (the upper part) and the gauging method (the lower part) for the interfacial undercooling.

The experimental platform we adopted is known as a horizontal Bridgeman directional freezing stage. This platform can avoid the convection interference caused by gravity. Furthermore, it aims at producing a constant and uniform thermal gradient along which samples of colloidal suspensions are pushed mechanically at a constant pulling speed.

The sample cell of colloidal suspensions (sample 1) is placed side by side with a cell of its solvent (sample 2) under an identical thermal gradient, shown as the lower part of Fig.S1. Interface gaps between two samples are recorded and measured through snapshot. The separation distance between melting front of the solvent (red dash line in Sample 2) and interface position of suspensions (blue dash line in Sample 1) indicates the interfacial undercooling of colloidal suspensions. Through the difference of pixels (ΔS) between these two lines on the photograph combined with image scale (M) and temperature gradient (G), the interfacial undercooling (ΔT) of colloidal suspensions is calculated as $\Delta T=S\times G$ ($S=\Delta S\times M$ is the real distance of the interface gap).

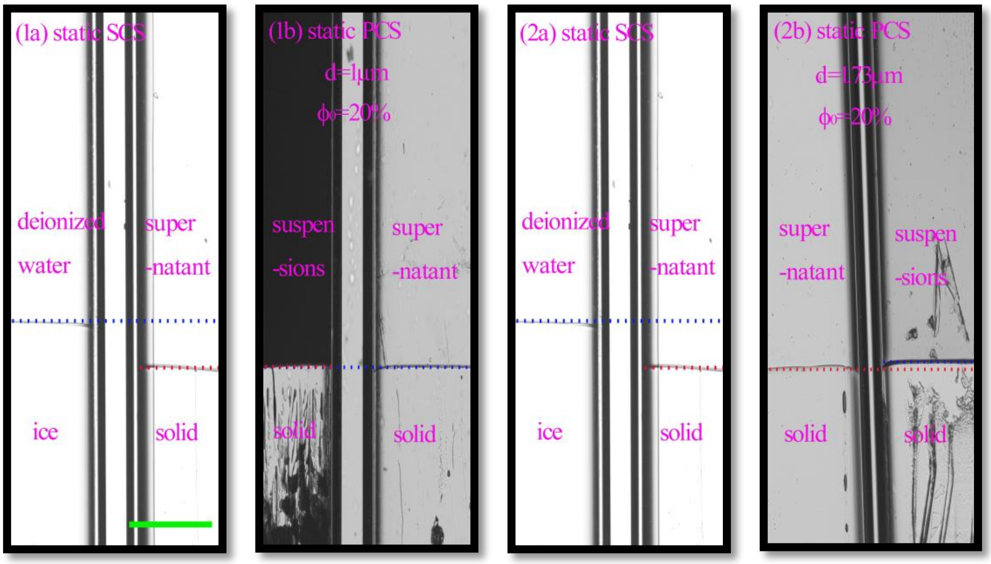

Fig.S2 The static interfacial positions in two side-by-side Hele-Shaw cells of the deionized water and the supernatant from PS colloidal suspensions with d=1μm and ϕ_0=20% (1a); and two side-by-side Hele-Shaw cells of the colloidal suspensions and its supernatant with d=1μm and ϕ_0=20% (1b); and two side-by-side Hele-Shaw cells of the deionized water and the supernatant from PS colloidal suspensions with d=1.73μm and ϕ_0=20% (2a); and the colloidal suspensions and its supernatant with d=1.73μm and ϕ_0=20% (2b) in a uniform thermal gradient of G=7.23K/cm. The distance of the static interfacial positions reveal the static interfacial undercoolings. The pulling speed V=0 and scale bar is 200 μm.

Figure S2 (1a) shows the measurement of static SCS for PS suspensions through the interfacial position comparison between the deionized water (left cell of Fig.S2 (1a)) and the supernatant (right cell of Fig.S2 (1a)). The discrepancy of the solid/liquid interface positions between the deionized water and the supernatant is 167.36μm, which indicates that the value of SCS is 0.121K in the consideration of G=7.23K/cm. Fig.S2 (1b) shows the measurement of static PCS for PS suspensions with d=1μm and ϕ_0=20%, through the interfacial position comparison between the suspensions (left cell of Fig.S2 (1b)) and its own supernatant (right cell of Fig.S2

(1b)). The interfacial position of the supernatant is almost parallel to that of its suspensions, which means that the freezing point of the supernatant is almost the same as that of its suspensions. Therefore, the PCS is undetectable and smaller than 0.01K, if it exists in this PS colloidal suspensions system. The results of Fig.S2 (2a) and (2b) for PS suspensions with d=1.73μm and ϕ_0=20% are similar to those of Fig.S2 (1a) and (1b).

Note that the brightness of Figure S2 (1a) is obviously higher than that of (1b). It is from the different light brightness of the microscope in different measurements. However, the interfacial positions are not affected by the different brightness from the optical microscope. We test different systems using the different colloidal suspension and its own supernatant by centrifugation each time. The measured data of PCS and SCS are shown in Table 1(a).

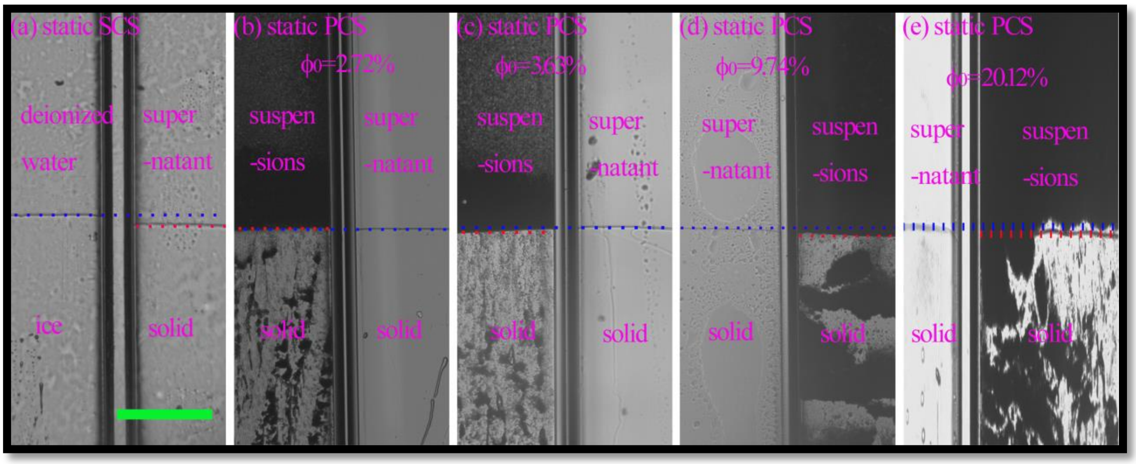

Fig.S3 The static interfacial positions in two side-by-side Hele-Shaw cells of the deionized water and the supernatant from alumina suspensions with d=50nm (a); and two side-by-side Hele-Shaw cells of the colloidal suspensions and its supernatant with ϕ_0=2.72% (b); and two side-by-side Hele-Shaw cells of the colloidal suspensions and its supernatant with ϕ_0=3.63% (c); and two side-by-side Hele-Shaw cells of the colloidal suspensions and its supernatant with ϕ_0=9.74% (d); and two side-by-side Hele-Shaw cells of the colloidal suspensions and its supernatant with ϕ_0=20.12% (e) in a uniform thermal gradient of G=7.23K/cm. The distance of the interfacial positions reveal the interfacial undercoolings. The pulling speed V=0 and scale bar is 200 μm. Note that each suspensions have been compared with their own supernatant. However the static interfacial positions of SCS for different suspensions are similar, and thus one static interfacial positions of static SCS is presented here to represent the four different static SCS.

Figure S3 (a) shows the measurement of SCS for alumina suspensions. The discrepancy of the solid/liquid interface positions between the deionized water and the supernatant is 55.60μm, which indicates that the value of SCS is 0.0402K in the consideration of G=7.23K/cm. Fig.S2 (b), (c), (d) and (e) show the measurement of PCS for alumina suspensions with ϕ_0=2.72%, 3.63%, 9.74%, 20.12% and d=50nm, respectively. Several measurements of these systems were taken and the average measured PCS for ϕ_0=2.72%, 3.63%, 9.74%, 20.12% and d=50nm are shown in Table 1(b).

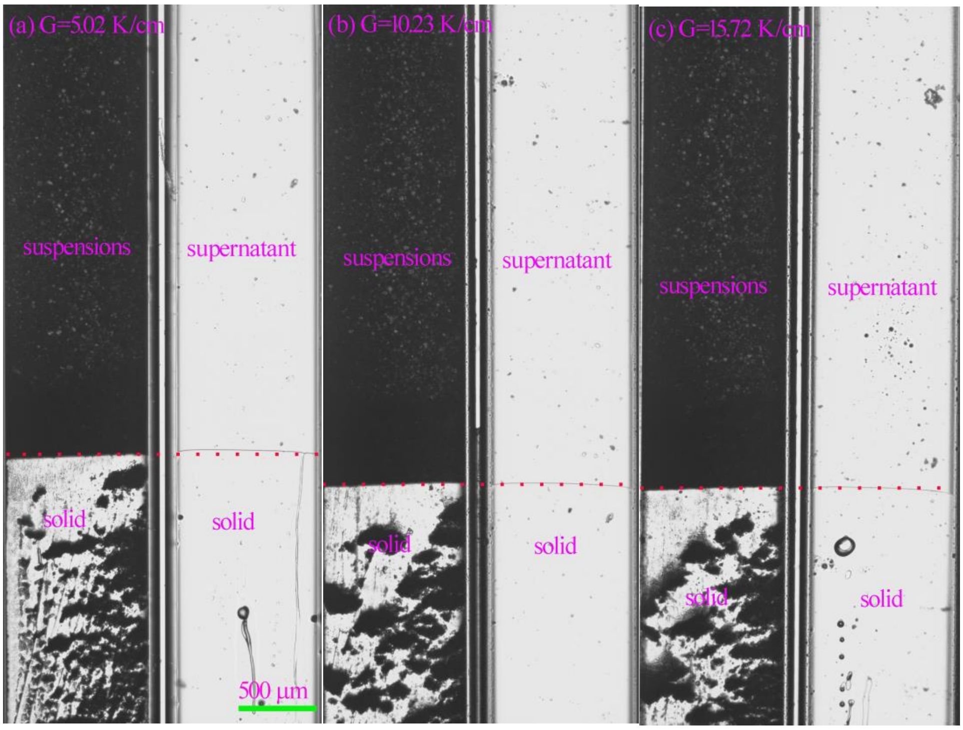

Fig.S4 The static interfacial positions in two side-by-side Hele-Shaw cells of the alumina suspension and its supernatant with ϕ_0=9.74% and d=50nm under different thermal gradients: (a) G=5.02 K/cm; (b) G=10.23 K/cm and (c) G=15.72 K/cm.

Fig.S4 (a), (b) and (c) show the measurement of static PCS for alumina suspensions with ϕ_0=9.74% and d=50nm under different thermal gradients: (a) G=5.02 K/cm; (b) G=10.23 K/cm and (c) G=15.72 K/cm, respectively. Although different thermal gradients are applied, the interfacial position (the red dot line) of the supernatant is always almost identical to that of its suspension, which indicates that a fixed temperature gradient is well imposed on these two adjacent cells. Thus the coincidence of interface positions can imply the coincidence of interface undercoolings.

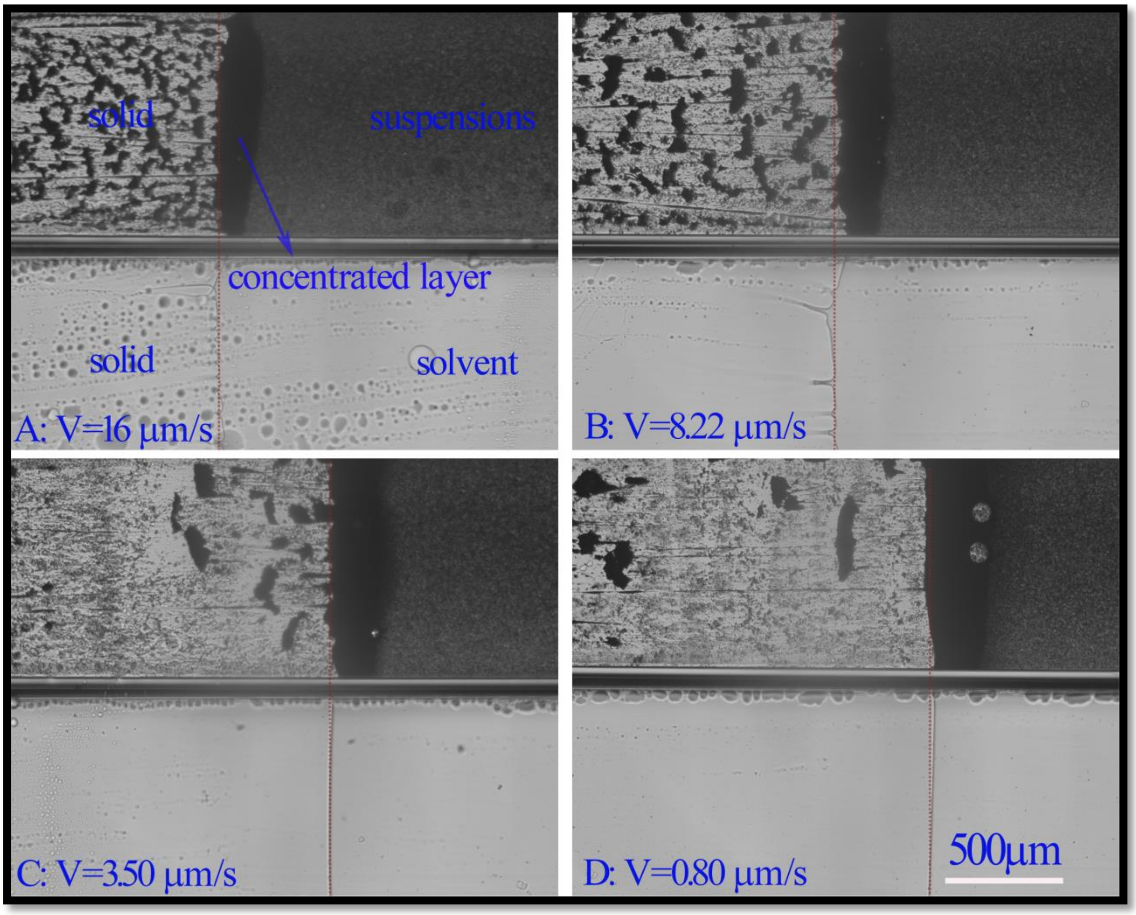

Fig.S5 the interfacial positions in two side-by-side Hele-Shaw cells of the deionized water and the supernatant (i.e. the solvent) from alumina suspensions with d=50nm and ϕ_0=3.63% under different pulling speeds, G=7.23K/cm.

Fig.S5 shows that the dynamic PCS for alumina suspensions under different pulling speeds were almost zero, although the particles were obviously accumulated in front of the advancing solid/liquid interface forming a clear concentrated layer. Accordingly, the concentrated layer seems invalid to cause an obvious PCS.

Although the bubbles formed in front of the advancing solid/liquid interface in Fig.S5 D, it is far away from the steady-state interface and doesn't affect the advancing interface as well as the dynamic PCS.

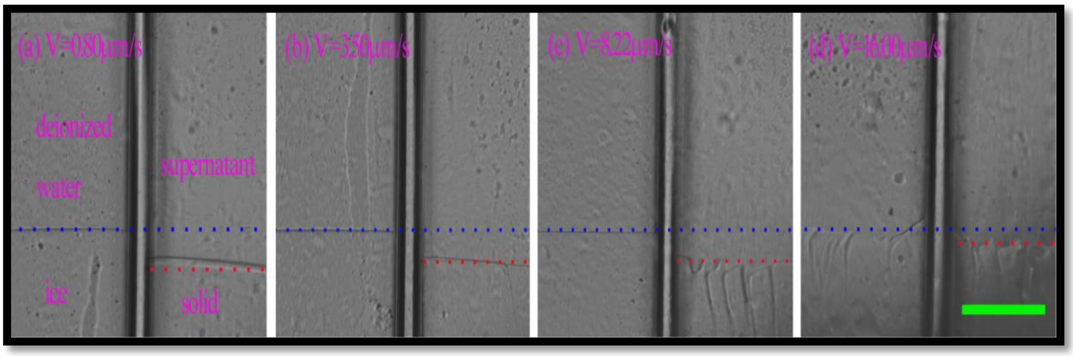

Fig.S6 the interfacial positions in two side-by-side Hele-Shaw cells of the deionized water and the supernatant of alumina suspensions under different pulling speeds and G=7.23K/cm, d=50nm, ϕ_0=3.63%. The scale bar is 200 μm

Fig.S6 shows the measurement of SCS for alumina suspensions. Left cell of each small picture is the deionized water and right cell of each small picture is the supernatant. The distance of the solid/liquid interface positions between the deionized water and the supernatant decreased with the increase of pulling speeds, which indicates that the dynamic SCS decreased with the increase of pulling speeds. The results are consistent with the classical alloy solidification principle [47].

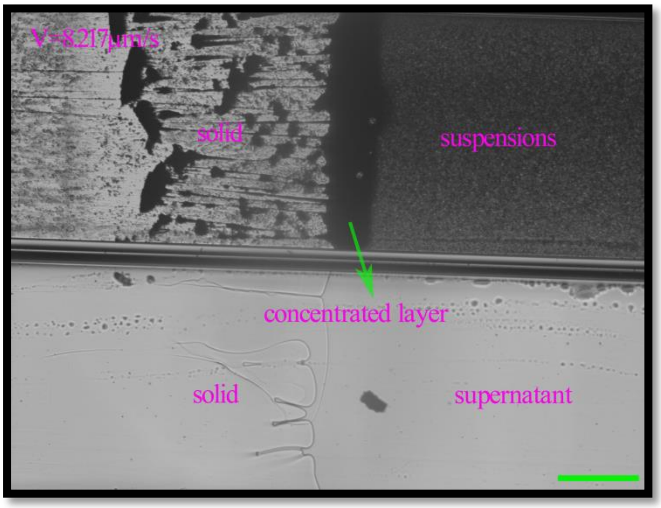

Movie S1 (Multimedia view) the steady dynamic interface position of PCS for alumina suspensions under V=8.217μm/s and G=7.23K/cm, d=50nm, ϕ_0=3.63%. The scale bar is 200 μm.

It is easy to check the steady dynamic interface. We kept track of the position of the interface through snapshot. If the interface doesn't move in the visual field, the interface position will be at steady state. Also the stability of the method for dynamic pulling test has already been verified by Ref.[37].

Although the bubble formed in front of the advancing solid/liquid interface, it is far away from the steady-state interface and doesn't affect the advancing interface as well as the dynamic PCS.
